# Studies of the Effectiveness of Bisphosphonate and Vanadium-Bisphosphonate Compounds* In Vitro* against Axenic* Leishmania tarentolae*

**DOI:** 10.1155/2016/9025627

**Published:** 2016-02-29

**Authors:** Amy T. Christensen, Craig C. McLauchlan, Anne Dolbecq, Pierre Mialane, Marjorie A. Jones

**Affiliations:** ^1^Department of Chemistry, Illinois State University, Normal, IL 61790-4160, USA; ^2^Institut Lavoisier de Versailles, Université de Versailles St-Quentin-en-Yvelines, 45 Avenue des Etats Unis, 78035 Versailles Cedex, France

## Abstract

Leishmaniasis is a disease that is a significant problem for people, especially in tropical regions of the world. Current drug therapies to treat the disease are expensive, not very effective, and/or of significant side effects. A series of alkyl bisphosphonate compounds and one amino bisphosphonate compound, as well as alendronate and zoledronate, were tested as potential agents against* Leishmania tarentolae*. Also, two polyoxometalates (POMs) with nitrogen-containing bisphosphonate ligands, vanadium/alendronate (V_5_(Ale)_2_) and vanadium/zoledronate (V_3_(Zol)_3_), were tested against* L. tarentolae* and compared to the results of the alendronate and zoledronate ligands alone. Of the compounds evaluated in this study, the V_5_(Ale)_2_ and V_3_(Zol)_3_ complexes were most effective in inhibiting the growth of* L. tarentolae*. The V_5_(Ale)_2_ complex had a larger impact on cell growth than either alendronate or orthovanadate alone, whereas zoledronate itself has a significant effect on cell growth, which may contribute to the activity of the V_3_(Zol)_3_ complex.

## 1. Introduction

Leishmaniasis is a disease caused by the* Leishmania* protozoan parasite that occurs in the tropical regions of Africa and Asia as well as Central and South America [1]. This disease is a significant problem for people in some 80–90 countries; it is estimated that 1.3 million new cases and 20,000–30,000 deaths from the diseases can be expected each year [1].* Leishmania* are parasitic trypanosomatids along with* Trypanosoma brucei* and* Trypanosoma cruzi* [2]. The three genera are a specific group of disease-causing kinetoplastid protozoa with a single flagellum. Kinetoplast protozoa are characterized by a single mitochondrion near the flagellum which contains DNA in a small compartment called a kinetoplast [2]. More than 20 different species of* Leishmania* can infect humans, and there are approximately 30 species of sand flies, the alternate host, that can spread the disease to humans. Other species of* Leishmania* can infect dogs, cats, goats, reptiles, and other animals [3]. Since the publication of the genomes of several species of* Leishmania* [4–7], there has been even more widespread study of the organisms and possible therapeutic avenues for leishmaniasis.

Drug therapies, including pentavalent antimonials, pentamidine (Nebupent*™*), amphotericin B (Fungizone*™*), and miltefosine (Miltex*™*), are currently employed to treat leishmaniasis, each with different mechanisms of action [8]. The pentavalent antimonial drugs are believed to inhibit parasitic glycolysis, fatty acid beta-oxidation, and ADP phosphorylation and their exact mechanism of activity is not known [8] although trypanothione* S*-transferase has been shown to play a key role [9, 10]. Amphotericin B is an antifungal agent causing parasitic cell lysis. Pentamidine interferes with the replication and transcription of genetic material in the parasite's mitochondria, and miltefosine is believed to disrupt parasitic cell surface receptors and change inositol, phospholipase, and protein kinase C metabolism [8]. These therapies are mostly given systemically and not topically. However, none of these drugs have been found to be satisfactory in meeting the needs of drug therapy, which are to be effective, economical, and have minimal side effects. Amphotericin B and miltefosine are expensive drugs and cause complications, and disease resistance to pentavalent antimonials is now widespread in India [11–14]. Development of better drugs for the treatment of leishmaniasis is needed because it would improve the quality of life for millions of people; thus new therapy targets should prove useful. One possible option is bisphosphonate compounds.

**Figure 1:**
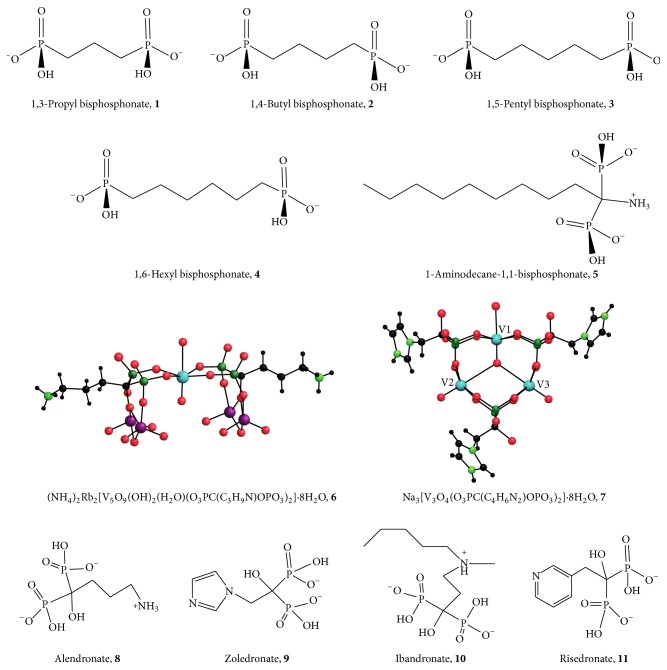
Bisphosphonate compounds. Compounds** 1**–**5** are expected to be doubly deprotonated at the experimental pH (7.5) resulting in a net negative charge.

Bisphosphonate compounds are reported to have antibacterial, herbicidal, anticancer, and antiparasitic properties, and they are also reported to be involved in the activation of T cells [15–17]. Bisphosphonates derived from fatty acids have been reported to adversely affect* T. cruzi* and other trypanosomatids [15, 16]. Therefore, evaluation of the effects of bisphosphonate compounds on* Leishmania* parasites is warranted. Docampo and Moreno [17] report that some bisphosphonate compounds inhibit the growth of trypanosomatid parasites including* Leishmania donovani* both* in vitro* and* in vivo*. Studies point to the ability of bisphosphonates to inhibit the farnesyl pyrophosphate synthase (FPPS) enzyme in the parasite's cytosol as their mechanism of activity [17].* Leishmania major* promastigotes, genetically modified to overexpress the FPPS enzyme, were less affected by the bisphosphonate risedronate, and the effect of the bisphosphonate on the parasite decreased as the enzyme's activity increased [17]. Several nitrogen-containing bisphosphonate compounds, namely, alendronate, zoledronate, ibandronate, and risedronate (Figure 1,** 8**–**11**), often in their acid form, are currently used to treat conditions including osteoporosis, Paget's disease, hypercalcemia, bone tumors, and other bone diseases [18]. These four nitrogen-containing bisphosphonate compounds are geminal bisphosphonates with a P-C-P backbone [19]. Bisphosphonates, then, are worth examining as antileishmanial therapeutic agents.

Metal complexes have also been examined as antileishmanial agents, including complexes containing vanadium [20–24]. Vanadium is a well-known phosphatase inhibitor [25–29], and we have previously examined phosphatase inhibition by vanadium complexes as an avenue for anti-*Leishmania* impacts by vanadium complexes [21, 22]. Given the effective metal-complexing behavior of bisphosphonates, some synergistic effects of bisphosphonates and metal complexes may be expected. For example, polyoxometalates (POMs; reviewed by [30]), anionic metal and oxygen clusters, are known to adversely affect tumors and viruses as well [15] and POMs of Mo^V^ or Mo^VI^ ion complexes with bisphosphonates have been studied against three different human tumor cell lines: MCF-7 (breast adenocarcinoma), NCI-H460 (lung large cell), and SF-268 (central nervous system glioblastoma) [19]. Compain et al. [19] found that a Mo^VI^-alendronate complex was most effective against all three lines and that the Mo-POM and bisphosphonate ligand have a synergistic effect. El Moll et al. [15, 31] found similar metal-bisphosphonate efficacy against the same cell lines in an extended study, with the most effective being V-zoledronate complexes.

Demoro et al. [32] reported that copper, cobalt, manganese, and nickel metal complexes with the bisphosphonate ligand alendronate or pamidronate were effective against* Trypanosoma cruzi* amastigotes, the trypanosomatid that causes Chagas disease [32]. Fernández et al. [33] found that V^IV^ complexes adversely affected* T. cruzi*, but V^V^ complexes were ineffective against the parasite. Their data also indicated that the effectiveness of the compounds was related to the stability of the V^IV^ complexes [33].

Docampo and Moreno [17] note the existence of a proton translocating pyrophosphatase (V-H^+^-PPase) enzyme localized in the acidocalcisome in some parasitic protozoa including* L. donovani* and* L. amazonensis* as another potential drug target for bisphosphonate compounds. The acidocalcisome is an organelle with a high concentration of calcium and phosphate ions found in pathogenic microorganisms, green algae and slime molds. Bisphosphonate compounds are analogs of pyrophosphates and have been shown to inhibit V-H^+^-PPase in mung bean plants [17]. Docampo and Moreno speculate that bisphosphonates targeting this enzyme activity could be a new direction in the treatment of leishmaniasis with bisphosphonates [17].

Here we report the effects of a series of four alkyl bisphosphonate compounds, one amino alkyl bisphosphonate compound, as well as alendronate (Ale) and zoledronate (Zol), on axenic* Leishmania tarentolae*. Two polyoxometalates (POMs), vanadium/alendronate ((NH_4_)_2_Rb_2_[V_5_O_9_(OH)_2_(H_2_O)(O_3_PC(C_3_H_9_N)OPO_3_)_2_]·8H_2_O,** 6**, V_5_(Ale)_2_) and vanadium/zoledronate (Na_3_[V_3_O_4_(O_3_PC(C_4_H_6_N_2_)OPO_3_)_2_]·8H_2_O,** 7**, V_3_(Zol)_3_), were also tested with* Leishmania tarentolae* and results were compared to those with the alendronate and zoledronate ligands alone. We expected to determine whether (or not) these bisphosphonate compounds would have a negative effect on the* L. tarentolae* parasite* in vitro*.

## 2. Materials and Methods

### 2.1. Chemicals and Materials

The following bisphosphonate compounds (Figure 1) were tested with axenic* Leishmania tarentolae* in culture: 1,3-propyl bisphosphonate (**1**), 1,4-butyl bisphosphonate (**2**), 1,5-pentyl bisphosphonate (**3**), 1,6-hexyl bisphosphonate (**4**) (**1**–**4** obtained from acids provided by A. Herlinger, Loyola University, Chicago, IL), and 1-aminodecane-1,1-bisphosphonate (**5**, commercially available from Sigma-Aldrich Chemical Co., St. Louis, MO). Sodium alendronate (Na**8**·3H_2_O, Alfa Chemical, Berkshire, England) and zoledronic acid hydrate (**9**, Santa Cruz Biotechnology, CA) were obtained from commercial sources. Vanadium POM bisphosphonate analogues V_5_(Ale)_2_ ((NH_4_)_2_Rb_2_[V_5_O_9_(OH)_2_(H_2_O)(O_3_PC(C_3_H_9_N)OPO_3_)_2_]·8H_2_O,** 6**) and V_3_(Zol)_3_ (Na_3_[V_3_O_4_(O_3_PC(C_4_H_6_N_2_)OPO_3_)_2_]·8H_2_O,** 7**) formed from alendronate or zoledronate, respectively, were synthesized as previously described [15].

### 2.2. *Leishmania tarentolae*

*Leishmania tarentolae* is a species of* Leishmania* parasitic protozoa that infects reptiles and has been shown to be a good model system for testing promising compounds that have anti-*Leishmania* activity [34], although there are differences in the genomes [7] and different species of trypanosomes are known to react differently to the same treatment [16].* Leishmania tarentolae* (ATCC 30143) have a predictable growth pattern which lends itself to visual observation of cell health. The parasites were grown at room temperature in 25 cm^2^ canted flasks (Corning, Inc.; Product number 430372) in sterile brain heart infusion (BHI) medium (BHI; Becton, Dickinson and Co., Sparks, MD; Product number 211059). BHI powder (18.5 g) was mixed with 500 mL of nanopure water and then autoclaved for 21 min. at 250°F and 20 pounds per square inch of pressure using the gravity setting. After autoclaving and cooling, 2 mL of 2.5 mM sterile hemin and 5 mL penicillin/streptomycin (10,000 units/mL and 10 mg/mL, resp.; Sigma-Aldrich Chemical Co., St. Louis, MO) were added to the growth medium using the standard methods of Morgenthaler et al. [35].

The parasites were observed microscopically to monitor the effect of each compound on the parasite. A Jenco International, Inc. (Portland, OR) inverted compound microscope Model CP-2A1 was used for microscopic evaluation of the parasite. The microscope could be adjusted to focus on cells at the bottom, middle, or upper parts of the culture flask which allowed observations of the parasite throughout the culture medium. Images of cells (at 400x magnification) were taken with a Kodak EasyShare C743 digital camera using close up or video mode. The spectrophotometric 3-(4,5-dimethylthiazol-2-yl)-2,5-diphenyltetrazolium bromide (MTT) (Product number M5655-1G, Sigma-Aldrich Chemical Co., St. Louis, MO) cell viability assay [36] was also used as a quantitative measure of cell mitochondrial activity and therefore indirectly measured cell growth. Sample absorbance at 595 nm was determined with an iMark microplate reader (BioRad Laboratories, Hercules, CA). The BHI growth medium alone was considered as a blank value subtracted from the sample absorbance (BHI and cells). Results are reported as corrected absorbance mean ± standard deviation (SD) for *n* = 4 replicates. Microscopic analysis of the cells for motility, shape, and clumping was also employed.

### 2.3. Sample Preparation of Test Compounds

The seven test compounds (Figure 1) were evaluated for their effect on axenic* Leishmania tarentolae* cells in culture using a uniform cell population. To help adjust for variations in cell number and viability, large cultures were grown in 500 mL of BHI growth medium. The cells were allowed to grow in a shaker incubator for three days at room temperature before distribution as uniform 10 mL aliquots to the Corning flasks. The test compounds were dissolved in 67 mM Tris-Cl buffer, pH 7.5, or dimethyl sulfoxide (DMSO) as indicated; typically a stock solution was prepared by dissolving material in appropriate solvent at 100x final desired concentration. Control cells from the same large culture were grown without added compound or with 1% (v/v) added DMSO to control for that solvent when required. Tested concentrations were based on preliminary studies with sphingomyelinase (not shown) for** 1**–**5**. Complex** 5** was ineffective at lower concentration [37] so the concentration was increased to correlate with the work previously reported by Compain et al. with Ale [19]. Concentrations for POMs** 6** and** 7** were chosen based on averages of previously reported values [15].

Cells were incubated with each of the four alkyl bisphosphonates at a final concentration of 10 mM. Cells were also incubated with 1-aminodecane-1,1-bisphosphonate (**5** at 130 *µ*M final concentration), or cells were incubated with 1.0 mM final concentrations of the vanadium POM bisphosphonate compounds (**6**,** 7**). However, V_3_(Zol)_3_ (**7**) was not fully soluble in buffer or medium even following sonication despite its solubility in water, warming to 37°C, and vortexing; therefore, the actual solution concentration in the experimental flasks was* lower* than 1.0 mM, but we report the maximum possible value to show the effectiveness of the complex. Seven blank samples, one for each compound, were also prepared by adding the same experimental concentration of each compound to 10 mL of sterile BHI with no cells. For the 1-aminodecane-1,1-bisphosphonate compound (**5**), DMSO alone was added to the blank. The blank for the control cells was BHI growth medium.

The pKa values for two of the alkyl bisphosphonate compounds, 1,3-propyl bisphosphonate and 1,4-butyl bisphosphonate [38], indicate that the molecules are predominantly doubly deprotonated at the experimental pH level of 7.5, and it is expected that 1,5-pentyl bisphosphonate and 1,6-hexyl bisphosphonate are also doubly deprotonated under our experimental conditions.

## 3. Results

### 3.1. Importance of Standard Sampling Conditions via Examining Effect on* L. tarentolae* of 1-Aminodecane-1,1-bisphosphonate

**Figure 2:**
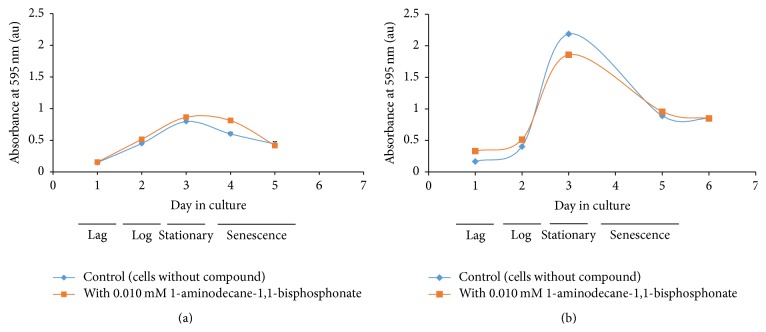
Cell growth curve (absorbance using MTT reagent versus day in culture) of* Leishmania tarentolae* with and without 1-aminodecane-1,1,-bisphosphonate (**5**). (a) and (b) are data from two representative experiments. Compound is added on day 2.

The importance of conducting all tests on uniform batches of cells is illustrated in the growth curves of* Leishmania tarentolae* incubated with and without 1-aminodecane-1,1-bisphosphonate (**5**), a known inhibitor of a phosphodiesterase enzyme and reported to inhibit enzymes involved in membrane phospholipid turnover [39, 40]. Data for two separate experiments are shown in Figures 2(a) and 2(b). For each experiment, the compound was dissolved in 100% DMSO and was added to one flask to give a final concentration of 0.01 mM compound and 1% DMSO. In another flask, cells were grown without the compound with an equal amount of DMSO. The growth curve data suggest that with the added compound (on day 2 of culture) the culture does not result in the same number of viable cells at stationary phase because the maximum absorbance value is lower and the apparent time to stationary phase differs. However, the log phase growth in both cultures appears comparable because the slopes of the growth curve during log phase are similar within and between experiments. Also, the culture with the added compound appears to undergo senescence sooner, suggesting that cells are not dividing or are dying more readily. This experiment was repeated several times, and all time growth curves of cell cultures with and without the compound were plotted. The four replicate experiments resulted in results which were not significantly different (using Student's *t*-test). The mean slope of the log phase of growth with the compound was 1.37 ± 0.81 relative to 1.07 ± 0.54 without the compound whereas the mean maximum absorbance value at 595 nm (A_595_) with the compound was 1.38 ± 0.41 relative to 1.52 ± 0.64 without the compound. Thus we conclude that this compound had little effect on the* Leishmania* culture, while also determining that different cultures produce difference cells in all phases of growth. To reduce cell culture variability, subsequent work was all completed with single batches of cell cultures and day 3 aged cells were used for subsequent experiments.

### 3.2. *L. tarentolae* and Test Compounds** 1**–**3** Hours after Compound Addition

**Figure 3:**
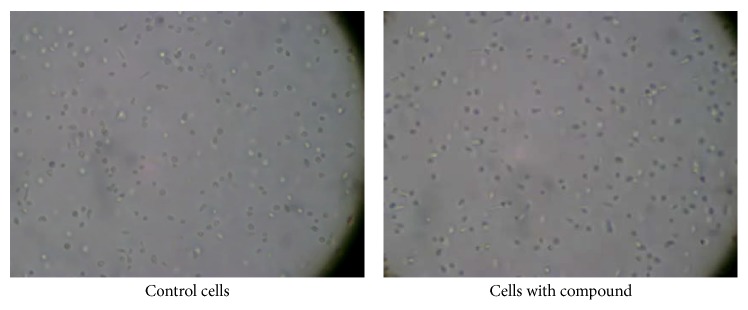
*Leishmania* cells one hour after 10.0 mM 1,4-butyl bisphosphonate addition (**2**) (400x).

A 500 mL batch of cells was prepared and on day 3 of culture the batch was separated into equal volumes in flasks, and different test compounds (**1**–**7**) were added to different flasks. Concentrations of intervention compounds were chosen based on preliminary results, as described in Section 2.3. The cells were examined microscopically within one hour after compound addition. In all of the experimental flasks the cells appeared to have much lower motility compared to the control cells; this reduction in motility leads to more cells found on the bottom of the flask. The cells in the control flask were active and undulating; thus fewer cells end up on the flask bottom.  Figure 3 shows control cells and cells incubated with 10.0 mM 1,4-butyl bisphosphonate (**2**). A noticeable motility difference is not obvious in these still photographs; however, these pictures are part of a video file in which the difference in motility was very obvious (see Supplementary Videos in Supplementary Material available online at http://dx.doi.org/10.1155/2016/9025627; control cells or cells 1 hour after addition of 1,4-butyl bisphosphonate).

**Figure 4:**
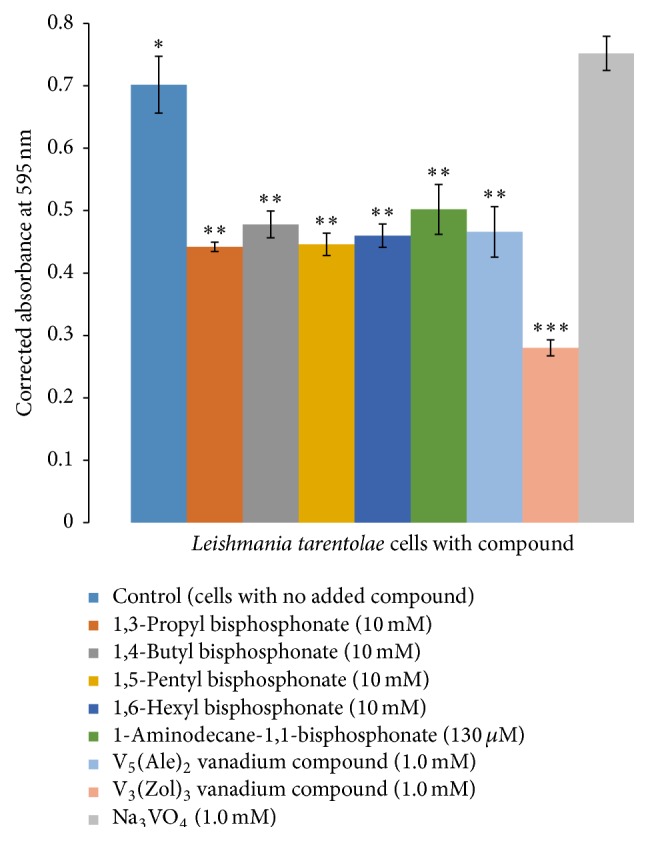
MTT cell viability assay of day 3 cells incubated 3 hours with test compounds (mean ± SD, *n* = 4).

Three hours after compound addition, 1 mL samples from each flask and its companion blank flask were withdrawn for the MTT cell viability test.  Figure 4 shows the resulting corrected MTT absorbance values; additional results, including those as percent of control, are displayed in Figure S1. Samples from flasks with the alkyl bisphosphonates (**1**–**4**) had about 35% less absorbance than the control cells. A sample of cells with the 1-aminodecane-1,1-bisphosphonate (**5**) showed a 28% decrease in absorbance. A sample from cells incubated with V_5_(Ale)_2_ (**6**) had 34% lower absorbance than the control, and a sample from cells with V_3_(Zol)_3_ (**7**) had a 60% decrease in absorbance. Because the MTT assay indirectly measures active mitochondrial reductases to process the MTT reagent, these data suggest a rapid detrimental effect of the test compounds on the test cells. Student's *t*-test (*p* ≤ 0.05) performed with the data statistically distinguished three separate groups based on mean ± SD values. The control is group 1 (*∗*), six of the seven experimental compounds are group 2 (*∗∗*), and the last compound, V_3_(Zol)_3_ (**7**, Figure 1), is group 3 (*∗∗∗*). Data indicate that at three hours after compound addition there was a statistically significant decrease in corrected absorbance value relative to control cells with the V_3_(Zol)_3_ (**7**,Figure 1) treated cells being the most reduced (by some 60% relative to control).

### 3.3. *L. tarentolae* and Test Compounds 24–27 Hours after Compound Addition

**Figure 5:**
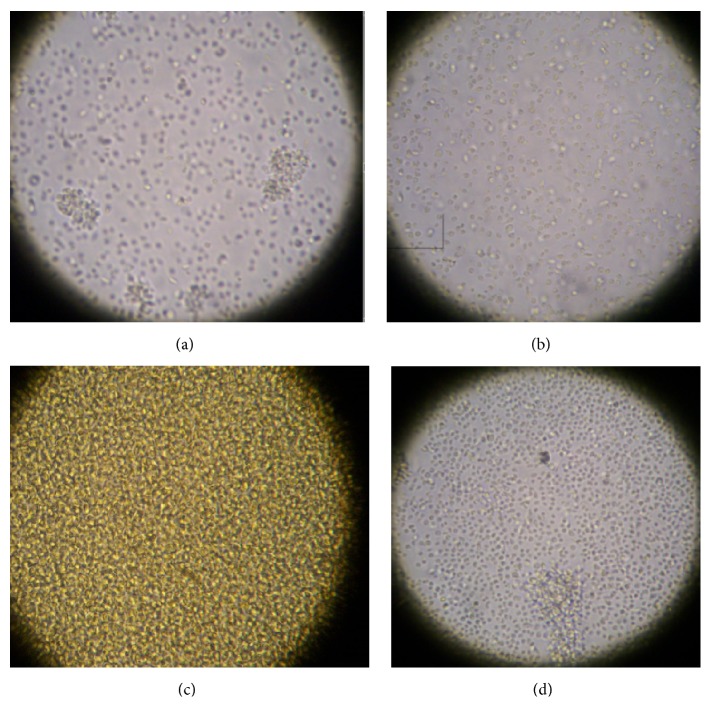
Microscopic observation of day 2 after addition of* Leishmania tarentolae* (a) control cells and cells incubated with representative test compounds (b) 1,4-butyl bisphosphonate (**2**, 10.0 mM), (c) V_5_(Ale)_2_ (**6**, 1.0 mM), and (d) V_3_(Zol)_3_ (**7**, 1.0 mM) for 24 hours (400x). See Figure 1 for structures.

On the second day of the experiment, the cell cultures (day 4 cells) were examined microscopically, and the cells in flasks with the alkyl bisphosphonates (**1**–**4**) were in poor condition (low motility and more circular shape) with many apparently lifeless cells on the bottom of the flask. The other samples appeared in varying degrees of distress.  Figure 5 shows the condition of the cells incubated with 1,4-butyl bisphosphonate (**2**), V_5_(Ale)_2_ (**6**), and V_3_(Zol)_3_ (**7**) (Figures 5(b), 5(c), and 5(d)).

The control sample shows clumping of cells, which is typical during the stationary and senescence phase of* Leishmania* growth [22]. Clumping occurs as the cell population increases causing depletion of nutrients and build-up of waste products. The cells incubated with these test compounds showed much less clumping, suggesting that cells are not responding normally. The cells incubated with compounds are more round than the control cells, which also suggests less cell vitality.

**Figure 6:**
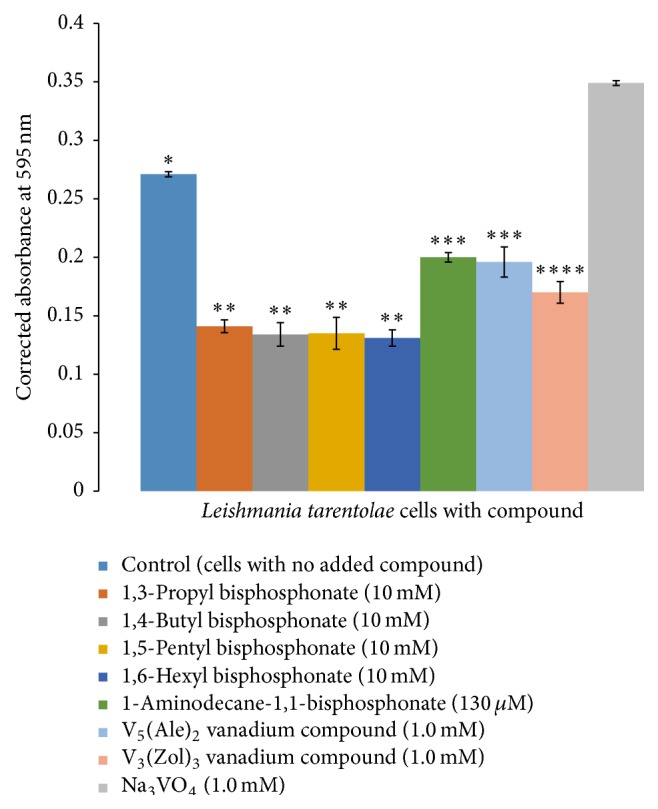
MTT cell viability assay of cells incubated with test compounds after 27 hours (mean ± SD, *n* = 4).

One mL samples from each flask sample were withdrawn for viability analysis. At 27 hours after compound addition, MTT results showed that samples from flasks with the alkyl bisphosphonates (**1**–**4**) had approximately 45% less absorbance than the control cells. The samples from flasks with the 1-aminodecane-1,1-bisphosphonate (**5**) or the V_5_(Ale)_2_ (**6**) addition exhibited a 27% decrease in absorbance relative to control cells. The sample from cells treated with V_3_(Zol)_3_ (**7**) had a 37% decrease in absorbance compared to the control cells (Figures 6 and S2).

Student's *t*-test (*p* ≤ 0.05) statistically distinguished four separate groups: the control is group 1 (*∗*), the alkyl bisphosphonates (**1**–**4**) are group 2 (*∗∗*), the 1-aminodecane-1,1-bisphosphonate (**5**) and V_5_(Ale)_2_ (**6**) compounds comprise group 3 (*∗∗∗*), and the V_3_(Zol)_3_ (**7**) is group 4 (*∗∗∗∗*). Thus at 27 hours after adding the various compounds there was a statistically significant decrease in corrected absorbance value for all tested compounds relative to the same age control cells. Cells with the V_3_(Zol)_3_ (**7**) compound appear to be able to recover from the effects of a single addition of this compound (from a 60% decrease in absorbance to a 37% decrease) in about 24 hours. However, this compound was not very soluble in either the buffer in which it was dissolved for the stock solution nor in the BHI cell medium. The solid compound was observed in the flask during the experiment suggesting that its effective concentration in the culture decreased as the experiment progressed. Therefore, this experiment does not accurately measure the concentration dependent effect of V_3_(Zol)_3_ particularly on* L. tarentolae*. Lower concentrations (100–200 *μ*M) were ineffective inhibitors of comparison enzymes [15], so they were not pursued further in this study. It appears that the other test compounds also adversely affect the parasite at the stationary and senescence stages because there is a significant decline in MTT response on experimental day two, which is day 5 of culture, typically shown as late in the culture growth phase.

### 3.4. Incubation of* L. tarentolae* with Alendronate, Zoledronate, and Orthovanadate

*L. tarentolae* were incubated with each of the ligands associated with the experimental vanadium compounds as well as the standard sodium orthovanadate, Na_3_VO_4_. Cells incubated with 0.02–2.0 mM alendronate (**8**) for 27 hours or zoledronate (**9**) at 0.03–0.3 mM for 27 hours exhibited no loss of cell viability or microscopic changes (data not shown). There was no significant difference between the cells grown without alendronate or cells grown with alendronate at concentrations of 0.02 mM, 0.2 mM, or 2.0 mM. The cells treated with 0.03 and 0.3 mM concentrations of zoledronate were not different from control cells after 27 hours of incubation. However, a decrease of approximately 50% in cell viability was observed with cells incubated with 3.0 mM zoledronate (**9**) for 27 hours as compared to the control cells. These data suggest that the adverse effects we observed on* L. tarentolae* after 27 hours of incubation with either vanadium bisphosphonate compound at 1.0 mM are due to the intact complexes** 6** and** 7** themselves and not due to dissociated ligands** 8** or** 9**, respectively.

### 3.5. Effects of Varying Dosages of 1,4-Butyl Bisphosphonate and V_5_(Ale)_2_ on Leishmania

**Figure 7:**
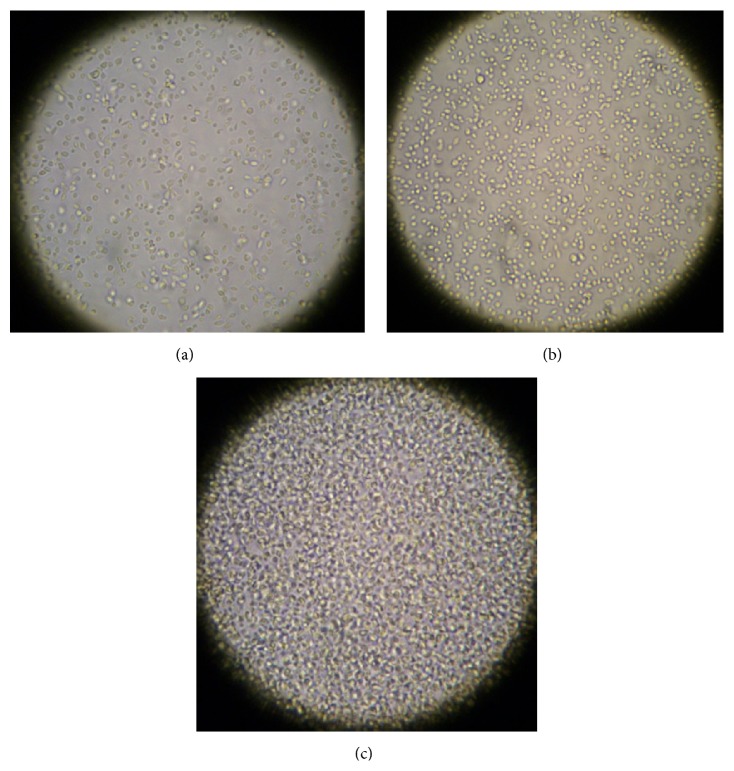
Microscopic evaluation of day 2 after addition of* Leishmania tarentolae* incubated with (b) 1,4-butyl bisphosphonate (**2**, 10 mM) and V_5_(Ale)_2_ (**6**, 1.0 mM) compared to (a) control cells (400x).

**Figure 8:**
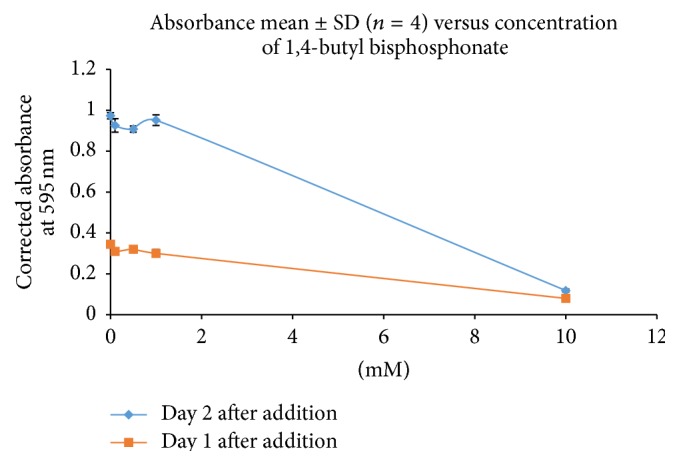
MTT cell viability of* Leishmania tarentolae* incubated with 1,4-butyl bisphosphonate (**2**, Figure 1) compared to control cells.

**Figure 9:**
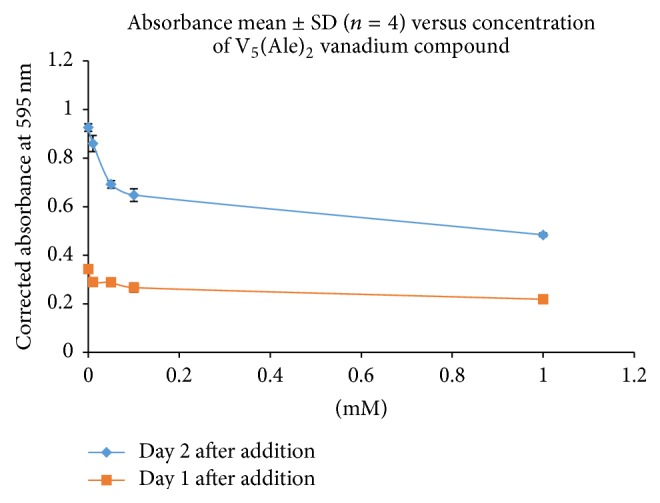
MTT data of* Leishmania tarentolae* incubated with V_5_(Ale)_2_ (**6**) compared to control cells.

As a representative sample two of the test compounds, 1,4-butyl bisphosphonate (**2**) and V_5_(Ale)_2_ (**6**), were selected to test in dose-response experiments with* L. tarentolae* using the cell batch method. Ten flasks were prepared for each compound: five with 10 mL of day 3 cells in BHI and five with 10 mL BHI for use as the appropriate blank. Compounds were prepared as earlier described, and concentrations of compounds (0.1–10.0 mM for 1,4-butyl bisphosphonate (**2**) and 0.01 to 1.0 mM for V_5_(Ale)_2_ (**6**)) were added to flasks with cells in BHI and the BHI only flasks. After the compounds were added, the cells were left undisturbed overnight. Samples from each flask were evaluated microscopically (Figure 7 and Supporting Video) and tested for MTT cell viability (Figures 8 and 9). Microscopically, the control cells appeared healthy with actively moving parasites; however, the cells with the highest concentration of 1,4-butyl bisphosphonate (**2**, Figure 1) on day 2 after addition (Figure 7(b) and Supporting Video) appeared round and stationary on the flask bottom which suggests cell stress and even death. The cells with the highest concentration of V_5_(Ale)_2_ (**6**) on day 2 after addition appeared dense and lifeless on the bottom of the flask relative to control cells (Figure 7(c)).

The MTT analysis of samples from flasks with 1,4-butyl bisphosphonate (**2**) on day one after addition (Figure 8) showed that those with the least amount of compound (0.1 to 1.0 mM) had MTT absorbance values statistically the same as the control cell absorbance. However, the MTT response from cells with the highest compound concentration of** 2** (10.0 mM) had an absorbance value 75% less than the control. On day two (Figure 8) after addition, the samples with the least concentrated amounts of compound** 2** again had absorbance values not different from the control sample, and cells in these flasks appeared to be growing and dividing like control cells. However, with the highest compound concentration of** 2** (10.0 mM), an absorbance value 90% lower than the control was measured which indicates that the cells have a greatly reduced viability.

On day one, the MTT absorbance of the samples incubated with V_5_(Ale)_2_ (**6**) at any concentration (Figure 9) was not different from the cells in the control flask. However on day 2, there was a more profound effect. Overall, the absorbance compared to the control was 46% less in cells with 1.0 mM of the compound, but there was also a marked decrease (30%) in absorbance, for cells with 0.1 mM of compound, compared to control cells. This suggests that V_5_(Ale)_2_ (**6**) affects the cells at a much smaller concentration than does 1,4-butyl bisphosphonate (**2**) as shown in Figure 8.

## 4. Discussion

Using axenic* Leishmania tarentolae* 1-aminodecane-1,1-bisphosphonate (**5**, Figure 1) was confirmed to be effective at reducing viability in this study; previously Roth et al. [40] had reported that this compound and other bisphosphonates prevented bacterial infections in rats. The alkyl bisphosphonates (**1**–**4**) at 10.0 mM concentration and vanadium POM compounds at 1.0 mM concentration (**6** and** 7**) were moderately effective at inhibiting both* Leishmania* motility and viability for at least 27 hours after the addition of a single dose to cultures of cells in log phase of growth. Both microscopic observations and viability tests indicate negative effects with a single dose on axenic cells. These compounds then may represent new therapeutic directions for* Leishmania* work. However, at this time, the mechanism of activity has not been established.

Compain et al. [19] tested POMs of Mo^V^ or Mo^VI^ ions and bisphosphonates on three different human tumor cell lines and found that the compound with the lowest IC_50_ (10 *µ*M) for all three types of tumor cells was a compound with a Mo^VI^ ion and alendronate ligands. Because the alendronate ligands alone had an average IC_50_ of 160 *µ*M when tested on the tumor cells, this suggests that the POM and the alendronate ligands together have a synergistic effect on the tumor cells [19, 31].

The two vanadium POM compounds tested in this study, V_5_(Ale)_2_ (**6**) and V_3_(Zol)_3_ (**7**), have been tested on three tumor cell lines by El Moll et al. [15]. The cell lines are MCF-7 (breast adenocarcinoma), NCI-H460 (lung large cell), and SF-268 (central nervous system glioblastoma). The V_5_(Ale)_2_ (**6**) compound averaged an IC_50_ of 0.6 *µ*M or a 1.2 *µ*M IC_50_ value per one bisphosphonate ligand for the three cell lines. The V_3_(Zol)_3_ (**7**) compound IC_50_ for the three cells lines was 0.3 *µ*M or a 0.9 *µ*M IC_50_ value per one bisphosphonate ligand [15]. In comparison the alendronate (**8**) and zoledronate (**9**) ligands alone when tested by El Moll et al. averaged an IC_50_ value of 150 *µ*M and 9.4 *µ*M, respectively, per compound which is also the per bisphosphonate ligand value. These data as well as the high activity of a fully inorganic compound suggest synergy between vanadium and ligands are less obvious than for the Mo compounds [15, 31]. El Moll et al. also note that they tested vanadium, molybdenum, and tungsten POMs, but the vanadium POMs had the lowest IC_50_ values [15].

Speciation of metal complexes, especially with vanadium [41–43], has been shown to be critical in efficacy of the active species [28]. Interactions of the vanadium with buffers, metabolites, substrates, and enzymes in assay conditions are also well established [44–48]. As such, studies with the POM, VO_4_^3−^, and the ligands individually were performed in each case. Our data show that the alendronate (**8**) ligand alone did not affect the growth of* Leishmania tarentolae* at the experimental concentrations of 0.02, 0.20, or 2.0 mM. The vanadium/alendronate compound, V_5_(Ale)_2_ (**6**), however, adversely affected* Leishmania tarentolae* at a concentration of 1.0 mM after three hours incubation with the compound; there was a 34% decrease in absorbance compared to control cells (Figure S1). This effect was also seen after 27 hours with the compound; the cells incubated with V_5_(Ale)_2_ (**6**) had 27% less absorbance compared to control cells (Figure S2).

The zoledronate (**9**) ligand alone was ineffective at 0.03 to 0.30 mM concentrations, but it did adversely affect the* Leishmania* cells when incubated at 3.0 mM. The absorbance values of cells incubated with 3.0 mM zoledronate for 27 hours were 50% less than control cells. After three hours of incubation with the POM V_3_(Zol)_3_ (**7**), the cells had 60% less absorbance than control cells, and the experimental cells had 37% less absorbance than control cells after 27 hours of incubation with the compound. However, because of the lack of solubility of V_3_(Zol)_3_ (**7**) in cell growth media, the compound could have more adverse effect on the cells than the data indicate.

It is not surprising that zoledronate (**9**) alone at 3.0 mM concentration had an adverse effect on* L. tarentolae* because zoledronate has been reported to be a potent compound. As reported previously, amino bisphosphonates such as alendronate (R = (CH_2_)_3_NH_3_^+^) are 10–100 times more potent in clinical use than when the R group is CH_3_. However, bisphosphonates with a nitrogen atom in a heterocyclic ring such as zoledronate (R = CH_2_(N_2_C_3_H_3_)) are up to 10,000 times more potent than when R is a methyl group [15].

The difference in potency reported here also is reflected in the dosage of two drugs used to treat osteoporosis. The Physician Desk Reference reports that, in humans, a daily dose of 10 mg alendronate sodium (Fosamax®) is used to treat osteoporosis [49]. In contrast, a 5 mg yearly dose of zoledronate (Reclast®) is used to treat osteoporosis in a human [50].

## 5. Conclusions

A series of alkyl bisphosphonate compounds and one amino bisphosphonate compound, as well as alendronate and zoledronate, were tested as potential agents against* Leishmania tarentolae*. Given the potency of both zoledronate and the V_3_(Zol)_3_ compounds as well as the difficulty in solubilizing the V_3_(Zol)_3_ in aqueous solutions, we speculate that, if safety and efficacy tests warrant, this POM compound may be effective in a skin cream formulation as a weekly or daily treatment for cutaneous leishmaniasis. This would allow more than a single dose to be easily applied. This is of importance since the majority of* Leishmania* infections are of the cutaneous type [51]. These compounds also have the added advantage of being quite stable at room temperature; thus easy storage and transportation to areas where infections by* Leishmania* are serious problems.

Future studies should examine the effect of the experimental compounds tested in this study on the proton translocating pyrophosphatase (V-H^+^-PPase) enzyme which is localized in the acidocalcisome of at least some of the species of* Leishmania* [17]. These bisphosphonate compounds, which are analogs of pyrophosphates, have been shown to inhibit V-H^+^-PPase in other organisms [17] and may be involved in the inhibition of the growth of* Leishmania* in these current studies.

## Supplementary Material

The Supplementary Material contains *Leishmania tarentolae* cell motility videos in the presence and absence of 1,4-butyl bisphosophonate, 2, and results of MTT cell viability studies at 3 and 27 h with complexes and controls.

## Abbreviations

Ale: Alendronate,** 8**
BHI: Brain-heart infusion
DMSO: Dimethylsulfoxide
FPPS: Farnesyl pyrophosphate synthase
MTT: 3-(4,5-Dimethylthiazol-2-yl)-2,5-diphenyltetrazolium bromide
POM: Polyoxometalate
*p*NPP: * para*-nitrophenylphosphate
PTP: Protein tyrosine phosphatase
SIM: Schneider's Insect Medium
TES: * N*-tris(hydroxymethyl)methyl-2-aminoethanesulfonic acid
Tris: tris(hydroxymethyl)aminomethane hydrochloride
V-H^+^-PPase: Proton translocating pyrophosphatase
V_3_(Zol)_3_: Na_3_[V_3_O_4_(O_3_PC(C_4_H_6_N_2_)OPO_3_)_2_]·8H_2_O,** 7**
V_5_(Ale)_2_: (NH_4_)_2_Rb_2_[V_5_O_9_(OH)_2_(H_2_O)(O_3_PC(C_3_H_9_N)OPO_3_)_2_]·8H_2_O,** 6**
Zol: Zoledronate,** 9**.
