# Supplementary material for: Studies of the Effectiveness of Bisphosphonate and Vanadium-Bisphosphonate Compounds In Vitro against Axenic Leishmania tarentolae
Source: Oxid Med Cell Longev. 2016 Feb 29;2016:9025627. doi: 10.1155/2016/9025627 (PMC4789522; doi:10.1155/2016/9025627)
Supplement: Supplementary file 1 — The Supplementary Material contains Leishmania tarentolae cell motility videos in the presence and absence of 1,4-butyl bisphosophonate, 2, and results of MTT cell viability studies at 3 and 27 h with complexes and controls. [file 9025627.f1.zip › 9025627.f1.pdf]

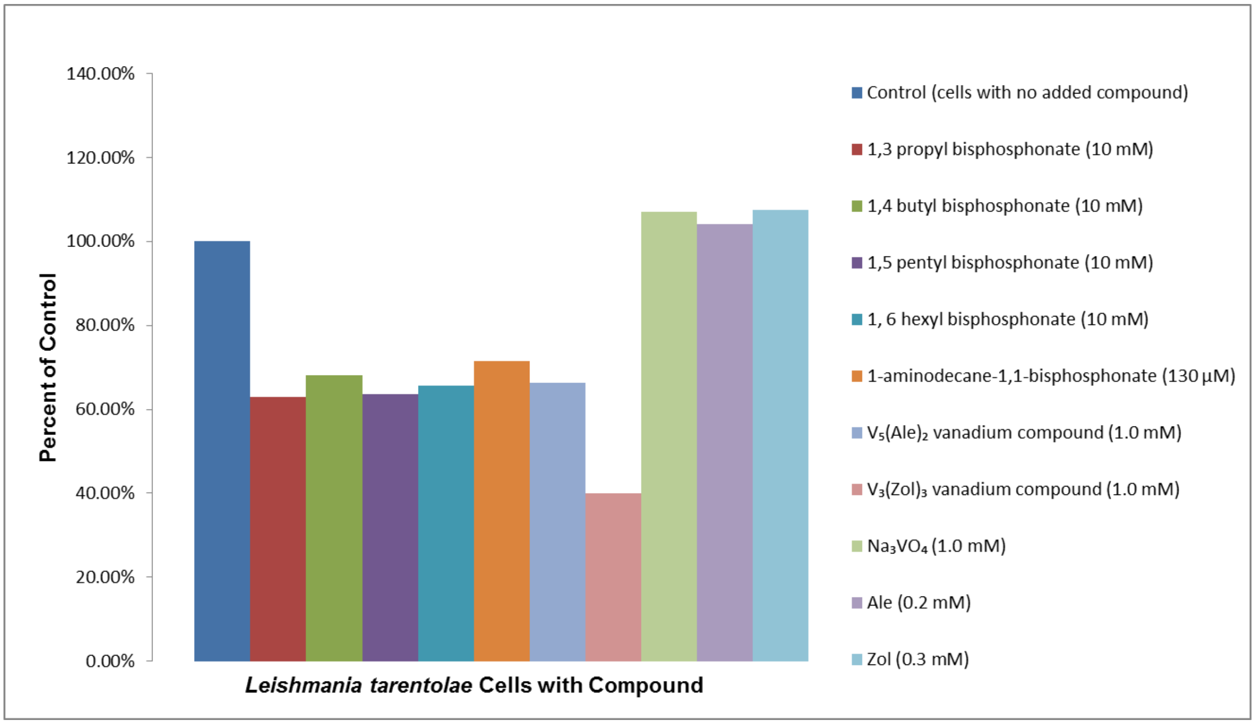

Figure S1. MTT cell viability assay of day 3 cells incubated 3 hours with test compounds (mean ± SD, n=4) as percent of control.

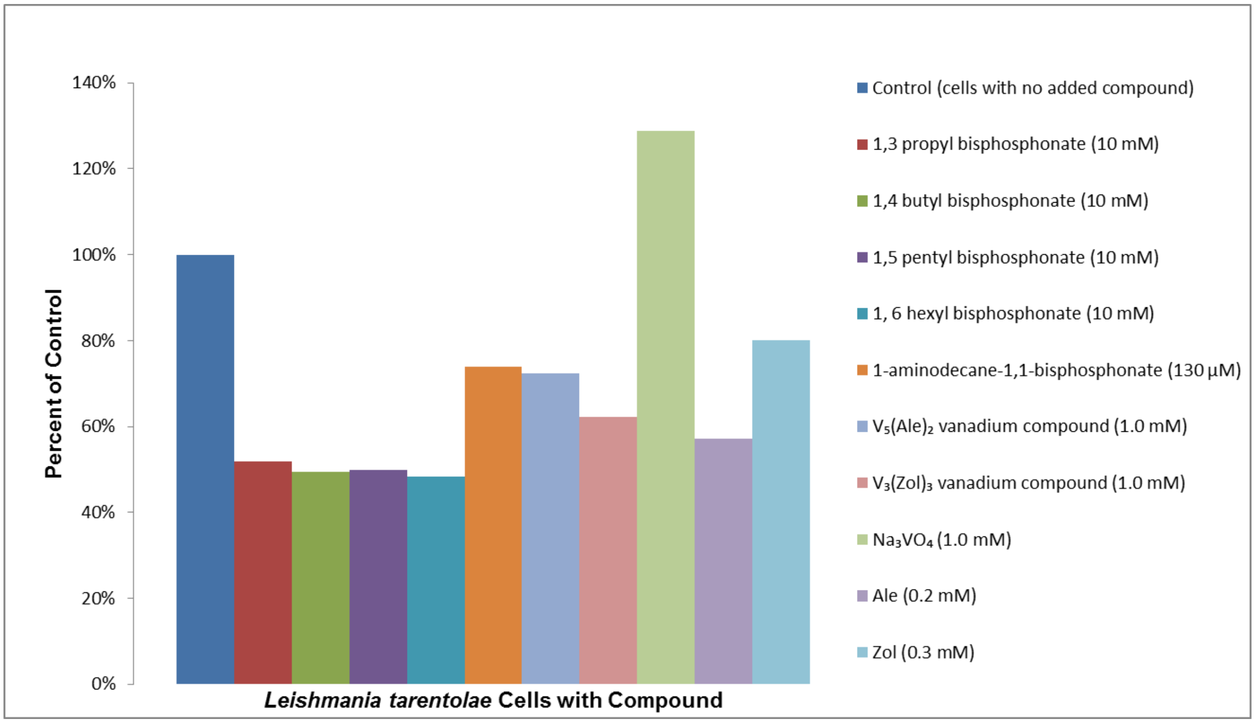

Figure S2. MTT cell viability assay of cells incubated with test compounds after 27 hours (mean ± SD, n=4) as percent of control.
